# Rethinking suicide prevention: from prediction to understanding

**DOI:** 10.1192/bji.2025.9

**Published:** 2025-11

**Authors:** Rachel Gibbons

**Affiliations:** MBBS, Royal College of Psychiatrists, London, UK.

## Abstract

Over the past two decades, suicide prevention efforts have expanded significantly, yet deeply held assumptions continue to shape policy in ways that may limit effectiveness. This paper critically examines key assumptions in suicide prevention, including the predictability of suicide, the role of suicidal ideation, and the conflation of self-harm and suicide. It challenges the view that mental illness is the primary cause of suicide and questions whether psychiatric hospital admission ensures safety. The paper also argues that overemphasis on prediction fosters fear-driven responses and explores how shifting the focus beyond risk reduction could foster more nuanced, compassionate and sustainable approaches to care.

## Introduction

Over the past two decades, suicide prevention efforts have expanded significantly and are likely to have contributed to a 36% global reduction in suicide rates. The field has also seen an extraordinary 8000% increase in publications (PubMed) since 1961. This progress has been linked to reduced stigma, public health interventions that limit access to means, increased research investment, and advances in data gathering and prevention strategies.^1^

Yet, alongside these advances, certain deeply held assumptions appear to shape both research and policy in ways that may constrain rather than enhance our understanding of suicide. Some beliefs – widely accepted but not always strongly supported by evidence – introduce bias into research,^2^ limit the effectiveness of interventions and discourage exploration of the complexity of suicide. This paper examines key assumptions in suicide prevention and advocates for approaches that acknowledge the complexity and unpredictability of suicide without overpromising what prevention can achieve.

## Can we really predict suicide?

Despite decades of research, our ability to predict individual suicides has not improved.^1,2,3,4^ Yet, belief in prediction persists, shaping discourse, research methods and conclusions. Suicidal behaviour arises from a complex interplay of genetic, psychological and social factors. Some acts are impulsive, others carefully concealed. Survivors of serious attempts often report uncertainty about their motives, many with no clear warning signs.^4^

If suicide is inherently unpredictable, does an overemphasis on prediction risk oversimplifying its complexity, hindering understanding and prevention, and paradoxically increasing ‘risk’ by fostering fear-driven rather than therapeutic responses?

## Suicidal ideation: a warning sign or something more?

Suicidal ideation is widely seen as a predictor of suicide, yet most who experience it do not die by suicide, and around 60% of those who do never expressed such thoughts. Its low positive predictive value underscores its limitations as a risk assessment tool^5^. Far from rare, suicidal ideation can be part of grief and mourning^6^ and reflects an ability to symbolise distress – a capacity often absent in those who die by suicide.^4,7^ When someone expresses suicidal thoughts, they are articulating somatic and psychic pain, offering a crucial opportunity for therapeutic engagement. Yet, again, anxiety-driven interventions can obscure this, prioritising immediate risk management over deeper understanding.

## Are self-harm and suicide two sides of the same coin?

Self-harm, which is common, and suicide, which is rare, are often mistaken as different expressions of the same issue. However, this misconception ignores their distinct functions, motivations and underlying dynamics, undermining the validity of research that examines them together.

Self-harm often acts as a coping mechanism – a way to express distress, regulate emotions or seek connection.^8^ Suicide, by contrast, typically reflects withdrawal from connection and an escape from unbearable psychic pain, often accompanied by fantasies of a pain-free existence after death.^5^ Although self-harm is the strongest predictor of future suicide, this may reflect shared mechanisms: the loss of mentalisation, the collapse of symbolic processing and the crossing of the body boundary, where psychic pain becomes physically enacted.^4,7,8,9^

## Does mental illness cause suicide?

The assumption that suicide is caused by mental illness has shaped prevention efforts, placing primary responsibility on mental health services, narrowing the scope of suicide prevention and leaving many deaths unexamined. In England, only 26–27% NCISH^10^ of people who died by suicide had contact with mental health services in the preceding year. Similarly, the CDC^11^ reported that 54% had no diagnosed mental illness. With one-sixth of the UK population experiencing mental illness at any time and 25% facing a common mental health problem each year, this overlap is unsurprising and inevitable in large-scale studies. Correlation may be mistaken for causation, reflecting Western assumptions that suicide is inherently irrational and tied to mental illness, with a focus on individual over social, cultural or spiritual factors.

Mental illness is often identified in those who die by suicide, but research relying on retrospective diagnoses is prone to bias and may overstate causation. Some suggest that mental illness may actually act as a defence against suicide, with risk increasing during recovery,^12^ and others propose that both mental illness and suicide may stem from an inability to mourn, with significant losses – bereavement, illness or relationship breakdown – often preceding both.^4,6^

## Does psychiatric hospital admission ensure safety?

It is widely believed that psychiatric admission ensures safety for those at risk of suicide, but evidence is limited. Large and Kapur in 2018 found only two studies on this and concluded that neither could determine whether hospital admission saves lives or increases suicide risk. Suicide rates during admission are estimated to be 50 times higher than in the general population, with post-discharge risk soaring to 133–300 times higher in the first month.^13^ Hospital admission may increase rather than reduce suicide risk. Large argues that restrictive environments, stigma and loss of autonomy can heighten distress, whereas Kapur suggests that the increased risk reflects a selection effect, as those admitted are already high-risk. However, both acknowledge that hospital admission itself contributes to post-discharge risk – Large attributing this to hospital conditions and Kapur to the sudden loss of structure and support, and feelings of abandonment.^13^ Institutionalisation may induce regression, weakening psychological defences and fostering dependency, thereby increasing vulnerability upon discharge. Individuals may feel exposed and unable to care for themselves until they regain internal resources. Given the strong influence of hospital safety on policy and practice, this warrants further investigation.

Removing ligature points is a key public health strategy in many countries, yet its impact in hospitals remains largely understudied. Ligatures are objects used for hanging or self-strangulation, whereas ligature points serve as anchors. In-patient environments contain many potential ligatures, making complete elimination nearly impossible (Table 1). Achieving this level of environmental control has required substantial resources, rebuilding and major changes to in-patient care. A cost–benefit analysis is needed to assess whether these measures improve safety or compromise the therapeutic environment. The failure to eliminate all risks has, at times, had serious organisational consequences.

**Table 1:** Ligature points and ligature materials in in-patient setting

Category	Examples
Doors and fixtures	Door handles, hinges, frames, automatic door closers
Windows	Window frames, window handles, restrictors
Furniture	Bed frames, shelving brackets, wardrobe rails, tables, chairs
Bathroom fixtures	Shower rails, towel racks, curtain rails, sink taps, exposed pipes
Medical equipment	IV stands, oxygen tanks, monitoring device cables
Handrails and support rails	Corridor handrails, grab rails in bathrooms
Lighting and ceiling fixtures	Ceiling-mounted lights, hanging light fixtures, smoke detectors, ventilation grilles
Electrical and communication equipment	Power cords, telephone cables, headphone leads, mobile phone charger leads
Curtains and blinds	Shower curtains, cubicle curtains, window curtains, Venetian blind cords or chains
Other exposed structures	Radiators, exposed plumbing, coat hooks
Clothing accessories	Belts, braces, laces, stockings, tights, bras, hoodie cords
Clothing items	Shirts, blouses, ties, trousers (which can be torn into strips)
Cords and ropes	Curtain pull cords, draw cords on bags, blind pull cords or chains
Plastic bags	Carrier bags, rubbish bags, clinical waste bags
Bedding	Bed sheets, blankets, pillowcases
Miscellaneous materials	Chains, hoses, rubber strips from fire doors or window seals

## Should suicide prediction and prevention be the primary job of mental health professionals?

Research shows that mental health professionals overwhelmingly believe that predicting and preventing suicide is a core part of their role, despite strong evidence that suicide prediction is not realistically achievable.^14^ This expectation is reinforced by societal pressures, which frame suicide as an outcome of mental illness, assuming it is both individually predictable and therefore preventable. Such beliefs fuel a search for blame when suicide occurs, leading to legal action, including manslaughter charges in some cases. This climate of fear and accountability risks distorting clinical priorities, diverting professionals from their fundamental role – not to predict the unpredictable, but, in line with other healthcare fields, to support physical, psychological and social health, promoting holistic recovery and improving quality of life.

## Can we truly keep people ‘safe’ from their own minds?

There is a widespread assumption that mental health services can keep those at risk ‘safe’. NHS England’s *Culture of Care Standards for Mental Health Inpatient Services* ^15^ references ‘safe’ 91 times, whereas the *NHS Patient Safety Strategy* ^16^ uses it 219 times. The frequent use of this term, often without clear definition, suggests an ideological rather than an evidence-based approach. But can we truly protect individuals from their own self-destructive impulses? Despite the best efforts of services, some remain determined and resourceful in ending their lives. Acknowledging this challenges the belief that suicide is always preventable and forces us to confront its inherent complexity.

## Can we reduce the suicide rate – even to zero?

The Zero Suicide Movement, originating from the Perfect Depression Care initiative of the Henry Ford Health System, gained momentum by promoting suicide as entirely preventable. In 2015, Mersey Care became the first UK mental health trust to adopt a zero suicide policy, leading to widespread National Health Service implementation. By 2018, the UK Government invested £2 million in a ‘zero suicide ambition’ for in-patients.

Although this the movement introduced valuable strategies – systemic accountability, risk screening and workforce training – its core concept remains problematic. Suicide is rare, complex, and shaped by historical, cultural and individual factors. Attempting to eliminate something not fully understood risks setting unrealistic expectations. Moreover, the implementation of zero suicide has placed an emotional burden on those bereaved by suicide, including clinicians. Framing suicide as entirely preventable can imply that every death is a failure, adding distress to caregivers. Although reducing suicide is crucial, acknowledging its complexity may foster a more compassionate, sustainable approach.

## Conclusion

The suicide prevention movement stands at a crossroads, navigating assumptions that may limit its effectiveness. These include the belief that suicide can be reliably predicted, the conflation of self-harm and suicide, the assumption that suicidal ideation leads to death and the conviction that mental health services ensure safety. Although well-intentioned, these perspectives risk oversimplifying a complex, deeply personal phenomenon shaped by cultural and social forces.

A focus on certainty and control risks reinforcing blame, fear and scapegoating rather than fostering understanding and support. Suicide remains complex and often enigmatic – acknowledging this is not failure but a step toward more meaningful, compassionate prevention. Clinicians, researchers, and organisations struggle with the tension between expectations and reality, pressured to provide certainty where uncertainty persists. But rather than viewing uncertainty as a barrier, could it be an opportunity? By broadening its focus beyond risk reduction to explore the existential, moral and philosophical dimensions of suicide, the movement can encourage deeper engagement with what it means to be human. Embracing the complexity of suicide may not only refine prevention but also deepen our understanding of suffering, resilience and meaning.

## Author biography

**Rachel Gibbons** is a consultant psychiatrist, psychoanalyst and group analyst. She works independently. She is chair of the Working Group on the Effect of Suicide and Homicide on Clinicians and the vice-chair of the Faculty of Medical Psychotherapy at the Royal College of Psychiatrists, London, UK.
